# Alcoholism and Drug Addictions

**Published:** 1910-06

**Authors:** W. A. Starnes

**Affiliations:** Atlanta, Ga.


### ALCOHOLISM AND DRUG ADDICTIONS.

BY W. A. STARNES, M. D., ATLANTA, GA.

In a recent number of *Success Magazine,* under the headlines of "Hope for the Victims of Narcotics," there appeared a lengthy article, signed by Dr. Alexander Lambert, portraying the discovery of a cure that will obliterate the craving for narcotics in the short space of from three to ten days. This is indeed something new, but we are much afraid that it is akin to the flashy advertising of some of the distinguished members of the medical profession. When a medical writer of reputation takes such a subject and discusses it for the laity, couched in terms of personal aggrandizement, it is easy to analyze the motive. It is surprising that medical journals could be misled into publishing such an article—it is regretible too, for while to the up-to-date informed physician the attempt will pass as a flimsy effort to impress an impossible procedure, such flashing statements have a harmful affect upon those physicians, and thousands of the public who take what they read from a doctor more or less for granted.

Dr. Lambert's experience may be of great value to himself, and to the medical profession, but it appears very strange to me, with my ten years of experience with the treatment of narcotics, that a man with his standing would publish such an article as he published in the November issue of *"Success."* He doubtless appreciates the great force of suggestion upon this class of patients, and with this fact in mind he attempts to victimize them.

In short, the article is a pseudo-scientific rehash of the effects of a well known remedy, with two practically inert preparations. Indeed, those who are familiar with the treatment of habitues of opium, cocaine or alcohol, will readily understand at a glance the feeble effort of Dr. Lambert.

The addict's physical condition from the chronic use of these narcotics is greatly impaired, and when one takes into consideration the pathological condition resulting from, perhaps, years' usage of these drugs, the assertion that one possesses a cure, the process of which requires but a few days, the statement is ridiculous upon its face. As a physician I regret such an attempt to commercialize practice as Dr. Lambert has made in his ridiculous, and positively non-scientific article. If Dr. Lambert possesses so great a discovery ( ?) the government should force him to empty our asylums, nervous and invalid sanitariums, and regenerate some of the most powerful intellects in our country.

Dr. Lambert states that his formula was given him by a layman, a certain Mr. Towns, and in this one respect we do not doubt him, for we can hardly conceive how one with even a semi-medical education would so mix drugs and label the mixture so startling a cure—but we marvel at the inconsistency of the doctor in stating that it will be necessary for the patient to be under the personal direction of Dr. Lambert. Surely, if a man like Mr. Towns, a layman without a medical education, could discover such a formula, patients with fair intelligence would not need a doctor for its administration, and certainly not so great a one as Dr. Lambert.

In the December issue of the same magazine there appears a statement that "letters by the hundreds have been received, and it requires extra help to answer. The people begging and pleading for relief." Such a statement itself is sufficient evidence of how easily the suffering victim is hood-winked and faked. My experience, based upon a close study in a sanitarium for ten or eleven years, seeing patients day and night, learning the characteristics and peculiarities, does not in the least coincide with the learned doctor.

I cannot see where Dr. Lambert claims to make such a SUCCESS with his formula, which is belladonna, xanthoxylum (or prickly ash), and hyoscyamus, with the proper amount of active catharsis to stimulate the liver and to produce rapid elimination. I would be glad to see a patient treated by this method and that patient cured in from three to ten days. You may find one patient in a thousand, or ten thousand, whom you can call cured in ten days—that is, you could have him off the drug, probably up and strong enough to take some exercise, eating fairly well and not taking any medicine of any kind—but that is only one in thousands.

My method of treatment is elimination from the very beginning, and after reducing the patient to a minimum dosage, (never allow your patient to become uncomfortable) then I administer, hypodermically, hyoscine—but not in large doses as was used at the beginning of this treatment. I endeavor to prevent a patient from having delirium, I have never yet gone so far as to produce unconsciousness, but only enough to carry the patient over that period of nervousness, which usually lasts from twenty-four to forty-eight hours, and sometimes longer, after the complete withdrawal of the narcotic. If you have had success and have been able to get the liver to acting, you have very little or no vomiting, but if you do you can usually wash the patient's stomach out, and one or two treatments usually eliminates the vomiting. Some patients are hungry and want to eat substantial food after you discontinue your hyoscine—to others, food is repugnant, and it is necessary to give them a liquid diet for a few days. You have to be careful to watch their diet or they will eat too much, and the system weak, the digestive organs haven't had time to resume their normal condition, and over-eating will often produce intense nervousness, and frequently other distressing symptoms.

It has always been my policy to treat each and every patient as their symptoms arise, and I think that is the only successful way to treat a patient. You cannot depend upon the hyoscine preparations exclusively, for frequently you will have a patient who has an idiosyncrasy to these drugs and you cannot give them with any degree of satisfaction, but such an idiosyncrasy usually manifests itself after a few doses.

The method of treatment that I have pursued in the last few years has been a most successful one, and a number of the most prominent physicians and surgeons in Atlanta can vouch for the success that I have had in treating and curing the drug and alcoholic diseases.

In the treatment of cocaine, very little treatment as far as drugs are concerned, is needed. What you want, is to retain your patient, keep him under constant surveillance for several months. This drug produces a very marked anemia of the brain, it produces insanity, and while you may take the drug away from the patient with very little ill affects and give him nothing afterwards, he will naturally crave it and want it, but he suffers practically no bodily harm. There is very little nervousness after leaving off this drug, and a patient can take it for days and nights, neither sleep nor eat, and if you take it away from him he will soon fall asleep and sleep from twenty-four to forty-eight hours and wake up refreshed, but if he has the drug he will immediately commence it again.

It is used in many different forms: hypodermically; sniffed up the nose; mixed with acetanilid; also the different nostrums of so-called catarrh cures. The sniffing of cocaine has become very common all over the country—in the south among the negroes, the lower class of white people and the sporting element, also among sailors and foreigners. It is very exhilarating at first, makes the person feel that they can do whatever they choose, have anything they want, or see anything they want to see. Their hallucinations are very acute.

The laws ought to be very strict. This drug ought not to be allowed sold under any circumstances except to physicians, and no physician needs to use but a very small amount of this drug during any one year—the nose and throat specialists, I think, use more of it than any other class of physicians—and legislation, conviction and punishment, in my opinion, is the only successful way of breaking up the cocaine habit.

In the treatment of alcoholic cases, Dr. Lambert seems to treat everything with the same drugs. I seldom use hyoscine in the treatment of alcoholic cases, although sometimes I do. When a patient comes in with delirium trements, or on the verge of them, I usually give a few doses to quiet the patient—and I use practically no opiate of any kind in the treatment of an alcoholic patient. I believe in elimination, and we must build the patient up with nerve sedatives, tonics, massages and electricity, and we must treat him from a suggestive and a moral point of view.

I can't help but feel that the article published by Dr. Lambert is more of an advertisement than anything else, and Dr. Lambert knows, as well as any man who has treated drug and alcoholic cases successfully, that he cannot cure them in from three to ten days, and if his patient is cured and ready to go home in from thirty to sixty days he should be well satisfied. And I emphatically repeat that you cannot successfully cure an average number of patients and discharge them as cured and ready to go home in less than thirty days—and that isn't really long enough, for your patient is not strong enough to go to work, or to get out into this world of hustle, bustle and excitement of to-day.

Just why Dr. Lambert cannot impart his treatment to well informed physicians, and why it is absolutely necessary for the patients to be under his personal direction, when he claims his treatment to be most simple and harmless, is hard to understand. Physicians, as a rule, can easily comprehend scientific instructions, and why a formula and outline, originated by a layman, can only be interpreted and demonstrated by one physician, at least, bespeaks an element of fraud.

At present in my institution there is a patient, a physician by the way, of culture and intelligence, who has recently undergone the Towns treatment with absolute failure. So in spite of the fancy tune of $350.00 in the hands of home operators, this one instance failed in such remarkable results as claimed in the Lambert article.

Whether the secular magazine received full advertising rates for the article in question, we have no way of determining. However, if to aid suffering humanity it was stimulated to publish such an article, we have grave fears as to the good accomplished.

